# Supplementary material for: What does it take to learn the rules of RNA base pairing? A lot less than you may think
Source: bioRxiv. 2025 Aug 2:2025.07.31.668042. Preprint. [Version 1] doi: 10.1101/2025.07.31.668042 (PMC12324431; doi:10.1101/2025.07.31.668042)
Supplement: Supplement 1 [file media-1.gz › supplemental_material/src/rscape_v2.6.0.tar.gz!/rscape_v2.6.0/lib/infernal/Manuscripts/inf-1-appnote/Proofs/Offprint.pdf]

OFFPRINT ORDER FORM

OXFORD JOURNALS
OXFORD UNIVERSITY PRESS

Fax: +44 (0) 1865 355741

This form should be returned to the above fax number

Bioinformatics

• MS number: to be quoted in all correspondence relating to your paper ....

A. FREE ONLINE ACCESS TO YOUR PAPER

Details of free online access to your paper will be sent to the corresponding author, who may then circulate them to co-authors.

Please note: owing to the resolution of online figures, colour and tone figures printed from pdf files may be of lower reproduction quality compared with those in publisher supplied printed offprints.

B. PURCHASE OF OFFPRINTS

Offprint Price List (£ Sterling UK; € Europe; US$ Rest of World)

No. of copies	1–8 pages			9–16 pages			Every further 8 pages		
	£	$	€	£	$	€	£	$	€
50	180	360	270	220	439	329	41	82	61
100	294	588	441	359	717	538	71	143	107
150	392	784	588	478	956	717	92	183	138
200	490	980	735	598	1196	897	112	224	168

Add 100% for any offprints including colour reproduction.
The above are author discount prices. For reprints with covers, industry reprints, reprints intended for commercial use or for quantities more than 200, please contact Oxford Journals for a quotation (email: special.sales@oxfordjournals.org)

I wish to purchase

………………………. offprints

ADDRESS FOR DELIVERY
(please print in capitals)

____
____
____
____
____

E-MAIL ADDRESS

____

C. PURCHASE OF SINGLE ISSUES

Copies of the issue in which your paper appears are available at a 50% discount on the single issue price (price information can be found on the journal web site).

I wish to purchase ………………….. copies of this issue

IMPORTANT

1. Cheques drawn on a UK or US bank should be made payable to Oxford University Press. Bank transfers of sterling (£) funds should be to Barclays Bank plc (bank sort code no. 20-65-18), PO Box 333, Oxford, OX1 3HS, UK, quoting account no. 70299332. Please quote full payment details. Full payment must be received by the publisher and you must therefore remit sufficient funds to pay all bank handling charges.
2. Orders from the EU: please supply details of your VAT registration or add local VAT (tax) to quoted prices if resident in: Belgium, Denmark, Finland, France, Germany, Ireland, Italy, Netherlands, Spain, Sweden. All other EU transactions are UK zero-rated.
3. Orders from Canada are subject to a Goods and Service Tax of 5%. This is not included in the prices given on this order form – please ensure payment includes this tax.
4. Offprints will not normally be mailed until the publisher is in receipt of either the appropriate payment or an official purchase order.
5. Please complete and return this form to Oxford University Press using the above fax number, along with the official order or the appropriate payment. Please keep a copy for your files. If your official purchase order is to follow at a later date, please return the completed form immediately, and mail a second copy with the official purchase order.
6. This form goes to you only; please ensure that the requirements of all authors are included in your order.
7. The above are pre-publication prices, and apply only to orders received before the publication goes to press. We suggest that you order your maximum requirements at this time, and remind you of the copyright nature of the material. For late orders the price will be given on application.
8. Offprints are supplied without covers in the regular size and format of the journal and only in the multiples shown above.
9. All orders beyond Europe are dispatched by air.
10. Claims cannot be considered more than three months after dispatch.

ADDRESS FOR INVOICE
(please print in capitals)

____
____
____
____

Tel:

☐ I wish to pay by MasterCard/Visa/ American Express/Switch
My account no. is

Expiry date ………………………………

If address registered with card company differs from the above please give details.

☐ Payment enclosed
☐ Please invoice

Signed …………………………………..

Date ……………… / ………. / ……....
